# The Role of Immune Mechanisms, Inflammatory Pathways, and Macrophage Activation Syndrome in the Pathogenesis of Hemophagocytic Lymphohistiocytosis

**DOI:** 10.7759/cureus.33175

**Published:** 2022-12-31

**Authors:** Omair Bseiso, Anas Zahdeh, Obay Isayed, Seewar Mahagna, Anan Bseiso

**Affiliations:** 1 College of Medicine, Hebron University, Hebron, PSE; 2 College of Medicine, An-Najah National University, Nablus, PSE; 3 College of Medicine, Al-Quds University, Jerusalem, PSE

**Keywords:** pathophysiology, macrophage activation syndrome, hlh, hemophagocytic lymphohistiocytosis, hyperinflammatory response

## Abstract

This review article describes the pathophysiology of hemophagocytic lymphohistiocytosis (HLH). The condition is characterized by excessive stimulation of inflammatory cytokines, lymphocytes, and macrophages, leading to hyperinflammatory disorder with immune dysfunction. The main clinical and diagnostic features include fever ≥38.5°C, splenomegaly, hyperferritinemia, cytopenia, hypofibrinogenemia, hemophagocytosis on the bone marrow, low or absent of natural killer (NK) cell activity, and elevated soluble CD25. Various immunological and inflammatory mechanisms are involved in the pathogenesis of HLH. Moreover, the condition can result in multisystem organ failure, contributing to the high mortality rate in hospital settings. A thorough literature search was conducted by collecting data from multiple articles published on PubMed, Medline, and Google Scholar. The article discusses the cellular and molecular pathways that lead to HLH. Due to the high rate of morbidity and mortality, early diagnosis needs to be established. More research pertaining to molecular biology, immunology, and the genetics of HLH is needed to explore the effective management and treatment of this rare disorder.

## Introduction and background

Hemophagocytic lymphohistiocytosis (HLH) is an aggressive and life-threatening syndrome characterized by increased levels of inflammatory cytokines and abnormal production of histiocytes resulting in multisystem organ failure [1-3]. This hyperinflammatory syndrome can occur in all ages, ranging from childhood to adulthood. The condition presents as a familial disorder (familial HLH, FHLH) or an acquired illness (secondary HLH, SHLH) in association with infection, malignancies, autoimmunity, and immune suppression. HLH can be diagnosed if at least five of the following characteristics are noted: fever ≥38.5°C, splenomegaly, increased ferritin levels, peripheral blood cytopenia, hypofibrinogenemia, hemophagocytosis on the bone marrow, low or absent of natural killer (NK) cell activity, ferritin >500 ng/mL, and elevated soluble CD25 (soluble interleukin 2 (IL-2) receptor alpha) [4,5]. It is a rare syndrome that is difficult to diagnose and treat because of the severity of the illness and the clinical overlap with other conditions such as DiGeorge syndrome, Kawasaki syndrome, hemolytic anemias, congenital liver cirrhosis, disseminated tuberculosis, and encephalitis. However, HLH, a life-threatening condition, can be prevented with early recognition and prompt treatment with supportive care [6]. Approximately 95% of children will die of the disease if left untreated [7], and the overall prognosis in adults is poor, with one large series showing a median survival of 2.1 months and an overall survival of about 34% at 42 months [8,9]. Treatment is directed at suppressing hypercytokinemia through immunomodulatory and immunosuppressive agents, cytostatic, T-cell, and cytokine antibodies [10].

HLH is characterized by hyperactive histiocytes and lymphocytes, which lead to a severely disturbed immune hemostasis. Different pathways are involved in the development of this rare disease. However, the exact mechanism behind the disease is not well understood. Moreover, due to the limited literature, non-specific signs and symptoms, and the low incidence of this disease, it continues to be diagnosed very late. Thus, patients with HLH possess a high mortality rate.

This review article highlights different mechanisms involved in the pathogenesis of HLH and addresses the immunologic mechanisms associated with HLH. It also demonstrates how a defect in immunologic cells can contribute to the disease and explains the role of different cytokines in inducing or halting inflammatory responses, which may lead to the disease. In addition, it illustrates the association between macrophage activation syndrome (MAS) and HLH. Finally, it recommends more studies of HLH to enhance the understanding of readers about molecular biology, immunology, and genetics of the disease and to explore more effective methods of diagnosis and treatment.

## Review

The research methodology comprises the data based on relevant abstracts, papers, and articles published in different journals on PubMed, Medline, PubMed Central, Google Scholar, Cochrane Library, and Medscape. First, the literature pertinent to the pathophysiology of HLH was thoroughly searched, and later the articles that focused mainly on the factors involved in the disease progression were included. Finally, the authors discussed the possible inclusion and exclusion of the articles. Therefore, articles strictly about human studies and clinical trials were included. Keywords of the review included “HLH,” “Hemophagocytic Lymphohistiocytosis,” “macrophage activation syndrome,” “pathophysiology,” and “hyperinflammatory response.” A total of 1,000 articles were initially reviewed, and 50 were finally selected based on their relevance to HLH and its pathophysiology. The search also focused on articles published in the last 10 years for the information to be as recent as possible.

Hemophagocytic lymphohistiocytosis associated with immunological mechanisms

One of the core immune defense mechanisms against infections with intracellular pathogens is contact-dependent cytotoxicity mediated by NK cells and cytotoxic T lymphocytes (CTLs). HLH can result from inborn defects in lymphocytes such as NK cells, CTLs, and T-regulatory cells, which generally mediate the control of infectious and inflammatory conditions within the immune system and in other tissues [11,12]. They are involved in the host defense mechanism against cancer and primary or secondary viral infections [13].

NK cells directly attack damaged or infected cells, independent of the major histocompatibility complex (MHC) class I. When NK cells are activated, they secrete perforin-containing cytotoxic granules and granzymes at the synaptic junction between cytolytic cells (NK cells and CTLs) and their target cells, leading to their lysis through caspase-dependent and caspase-independent apoptosis [12-16]. Therefore, early diagnosis of the NK cell defect is crucial for the prevention of life-threatening complications, as well as the implementation of necessary treatment [17].

Martinez et al. conducted a study with 20 patients to determine NK cytotoxic activity in patients with suspected HLH syndrome. In addition, they compared NK cell cytotoxicity with healthy controls according to age and sex. The study showed a significant decrease in NK cell activity compared with the controls (p = 0.001). Hence, the study helped elucidate an underlying mechanism involved in patients with primary HLH [18].

**Figure 1:**
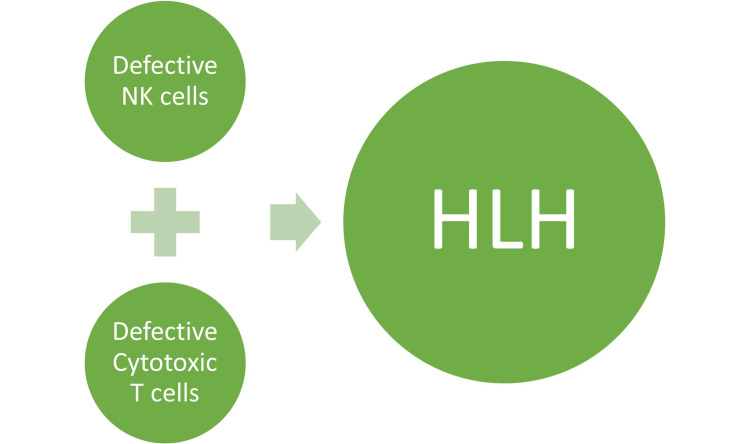
Impaired cytotoxicity interposed by NK cells and CTLs is directly involved in the pathogenesis of HLH. NK =  natural killer; CTL = cytotoxic T lymphocyte; HLH = hemophagocytic lymphohistiocytosis

The function of cytotoxic T cells is to kill autologous cells, carrying foreign antigens associated with the MHC class I. In contrast to NK cells, T cells recognize target cells using specialized T-cell receptors that bind specific MHC class I/peptide complexes on target cells. Therefore, CTLs build their immunosurveillance of intracellular homeostasis on MHC class I peptide presentation [19-21]. However, in HLH, genetic abnormalities lead to defects in proteins as well as ineffective antigen removal. This results in the disruption of immune surveillance and host defense systems. An alternative theory suggests that inefficient antigen clearance leads to persistent immunological activation and improper hemophagocytosis [21] (Figure 1).

Inflammatory pathway in the pathogenesis of hemophagocytic lymphohistiocytosis

HLH is a multisystem hyperinflammatory disorder characterized by the hyperactivation of T cells and macrophages. The clinical manifestations associated with this disease result from the overproduction of type 1 T-helper (Th1) cytokines, including interferon-gamma (IFN-γ), tumor necrosis factor-alpha (TNF-α), and IL-2, which leads to a chain reaction [22,23]. The defective T cells and unbridled macrophage activity lead to excessive cytokine production, subsequent immune dysregulation, and tissue damage [23].

**Figure 2:**
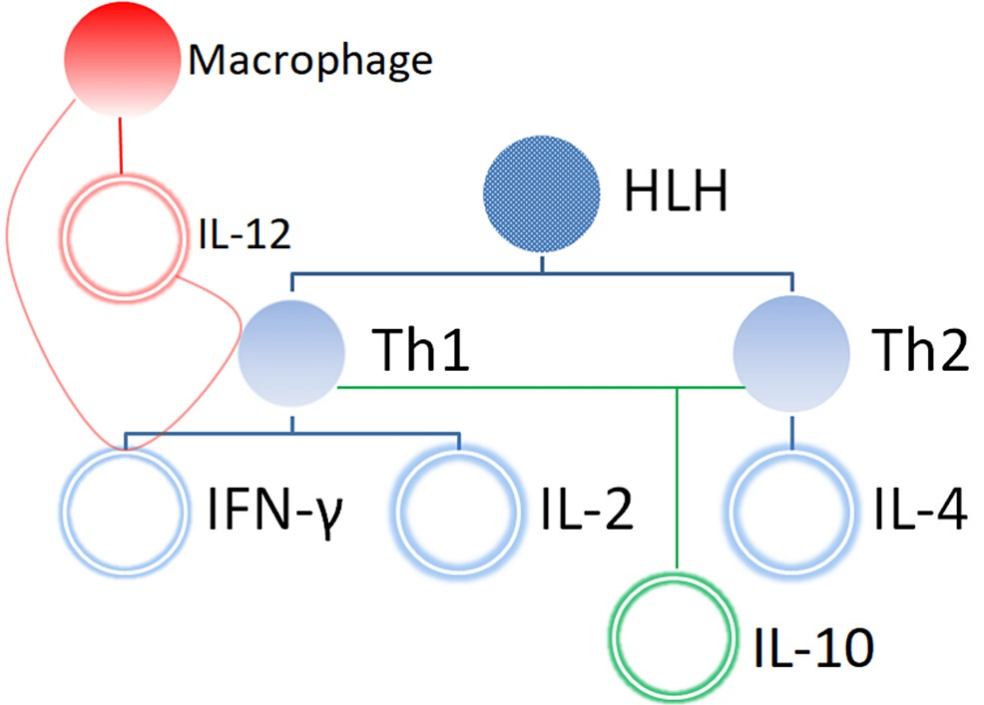
Th1, Th2, and macrophage paradigm illustrating IL-2, IL-4, IFN-γ, IL-10, and IL-12 production as a major pathway in HLH. Th1 = Type 1 helper; Th2 = type 2 helper; IL = interleukin; IFN-γ = interferon-gamma; HLH = hemophagocytic lymphohistiocytosis

The balance between Th1 and Th2 cells is determined by IL-12 and IL-4 [24-27]. B cells and macrophages produce IL-12, which favors the production of the Th1 response and induces IFN-γ [28-30]. Furthermore, IFN-γ enhances IL-12 production, leading to the production of more Th1 cells directly as well as endorsing the differentiation of Th0 cells and Th1 cells. IL-4 is another major Th2 cytokine, which is responsible for the formation of Th2 cells. IL-10, which is produced by both IL-12-induced Th1 cells and IL-4-induced Th2 cells, inhibits the production of IL-12, as well as other Th1 cytokines including IFN- γ and IL-2 [31-33]. The oppressive effects of IL-10 result in the inhibition of inflammatory cytokines and act as an anti-inflammatory agent [34]. Thus, IFN- γ, IL-10, 1L-4, and IL-12 are the major cytokines that play an important role in HLH (Figure 2).

Osugi et al. conducted a study with 11 patients with HLH to analyze the production of IL-12 and IL-4 responsible for activating the Th1 and Th2 response, respectively, and IL-10, which antagonizes the Th1 response. The results indicated that elevated levels of IL-12 and IL-10 were found in all patients with HLH, while IL-4 was not detected in any patients. Therefore, the study concluded that the production of cytokines prefers the development of Th1 cells over Th2 cells, and both cell types demonstrated a significant role in the pathogenesis of HLH. However, the overproduction of IL-10 was a suppressing way for the hyperactive Th1 cells and monocytes/macrophages in patients with this disease [35].

Macrophage activation syndrome

Usually, macrophages act as cells that present foreign antigens to lymphocytes for either direct destruction or the production of antibodies. Macrophages become activated and release cytokines in different types of HLH. In turn, when excreted in excess, cytokines can harm organs [36]. MAS is another fatal underlying mechanism involved in the pathophysiology of secondary HLH [36]. It is due to the overstimulation of macrophages which results in an overwhelming production of cytokines “cytokine storm.” It can be caused by rheumatic diseases such as systemic juvenile idiopathic arthritis and systemic lupus erythematosus. MAS manifests as high fever, lymphadenopathy, hepatosplenomegaly, hepatitis, cytopenias, elevated C-reactive protein, low erythrocyte sedimentation rate, hypofibrinogenemia, hypertriglyceridemia, and hyperferritinemia [37-40].

**Figure 3:**
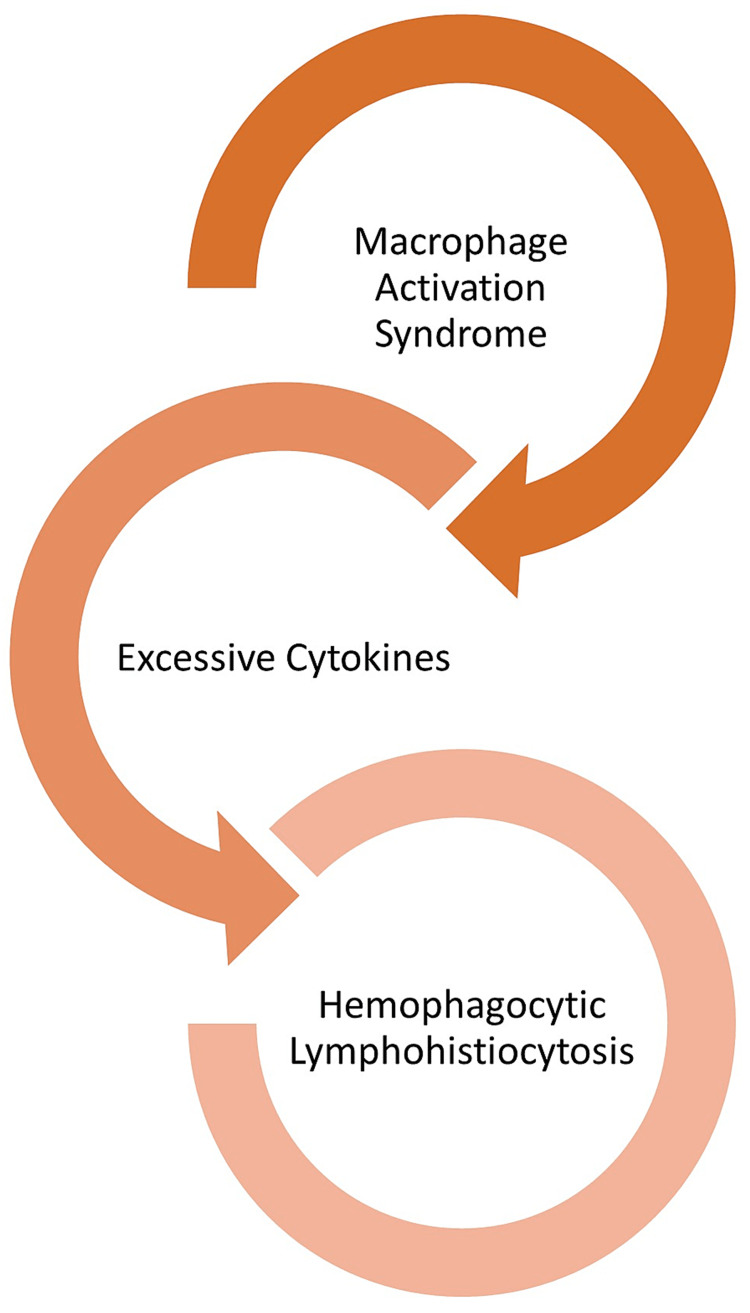
Uncontrolled activation of T lymphocytes and macrophages leading to MAS, which along with cytokine production is responsible for HLH. MAS = macrophage activation syndrome; HLH = hemophagocytic lymphohistiocytosis

Macrophages serve as antigen-presenting cells to present foreign antigens to lymphocytes for either direct destruction or antibody development. In HLH, macrophages become activated and secrete excessive amounts of cytokines, ultimately leading to severe tissue damage resulting in multisystem organ failure. Recent studies reveal the role of hypercytokinemia and hyperinflammation as the driving cause of pathology and morbidity/mortality in MAS [41,42] (Figure 3).

MAS is characterized by uncontrolled inflammation, which may lead to multiorgan failure and death in its severe form [43-48]. These manifestations are regulated by the substantial production of cytokines, which infiltrate all tissues and cause necrosis and organ failure. Because MAS can present in patients with autoinflammatory and autoimmune diseases, it should be carefully observed in HLH patients [49,50].

The types, pathogenesis, and diagnostic criteria of hemophagocytic lymphohistiocytosis

As mentioned earlier, HLH can be classified broadly into FHLH and SHLH based on the cause. The inheritance of defective genes such as *PRF1*, *UNC13D*, *STX11*, and *STXBP2 *contributes to the former, and the condition typically affects children and young adults, and the latter is associated most commonly with underlying infection or malignancy [3,6].

A multitude of clinical indicators and test results are used to make the diagnosis of FHL or SHLH. However, the clinical signs and symptoms could be more precise, and there is much overlap with other conditions. Thus, diagnosis is frequently delayed. The Histiocyte Society’s official diagnosis of HLH is based on meeting one or both of the following requirements: (1) molecular evidence supporting an HLH diagnosis. (2) Five of the following nine diagnostic standards for HLH: a fever, splenomegaly, cytopenias (affecting two or more of the three lineages in the peripheral blood), hypertriglyceridemia, hypofibrinogenemia, elevated ferritin, hemophagocytosis in the bone marrow, spleen, or lymph nodes, low or absent NK-cell activity, or elevated soluble CD25 (IL-2 receptor) [23].

Different theories have been proposed to explain the mechanisms involved in this rare disease and elaborate the diagnostic approach to this underdiagnosed illness [2]. Table 1 summarizes some of the relevant research studies that investigate these aspects of HLH.

**Table 1:** Relevant studies showing the mechanisms involved in HLH and some of the diagnostic approaches. HLH = hemophagocytic lymphohistiocytosis

Author name	Year	Journal	Aim of study	Main findings
Usmani et al. [1]	2013	British Journal of Hematology	To determine the advances in understanding the pathogenesis of HLH	This review article includes the factors affecting familial and acquired forms of HLH playing a major role in pathogenesis
Lehmberg et al. [2]	2013	British Journal of Hematology	To elaborate the current diagnostic approach to patients with HLH	This review article explains how HLH can be diagnosed and which crucial steps should be taken toward early management and treatment options
Zhang et al. [3]	2014	Cancer Control	To study the hereditary and acquired forms of HLH	This review article explains that the assessment of HLH can be done clinically and immunologically
Grzybowski et al. [4]	2017	Journal of Pediatrics Neurosciences	To understand the basic mechanism involved in HLH	This review article explains how early diagnosis and treatment can improve the survival of HLH patients
De Schuyter et al. [5]	2017	Acta Clinica Belgica	To evaluate the case of unexplained fever and pancytopenia	This case report includes how unexplained fever with pancytopenia can be one of the presenting features in HLH patients
Mehta et al. [6]	2013	Medical Oncology	The purpose of this review article is to explore the pathogenesis involved in HLH	This review article illustrates that HLH carries a high risk of mortality in primary and secondary types; therefore, early treatment is important for management
Rosado et al. [7]	2013	American Journal of Clinical Pathology	To understand the factors triggering both genetic and acquired HLH and their contribution to this rare disease	This review article focuses on new studies which are required to diagnose this rare disease and improve diagnostic methods and treatment
Filipovich et al. [11]	2015	Hematology/Oncology Clinics of North America	To determine the relation between inherited defects in immune cells and HLH	In this review article, it is explained that how genetic defects in cytotoxic cells and other immune cells play an important role in the pathogenesis of HLH
Kagi et al. [12]	1996	Annual Review of Immunology	To investigate the molecular mechanism of lymphocyte-mediated cytotoxicity	Review article focuses on cytotoxicity by CD4+ T cells and natural killer contributing to the immunological mechanism of HLH
Brisse et al. [13]	2015	Cytokines and Growth Factor Reviews-Journal	In this review, HLH was studied on animal models to explore findings on cell types, cytokines, and signaling pathways which are involved in disease pathogenesis	The aim of this review article is to show how genetic defects in granule-mediated cytotoxicity plays a major role in the immunological pathway of HLH
Weitzman et al. [14]	2011	Hematology. American Society of Hematology. Education program	What are the signs and symptoms leading to the diagnosis of HLH	This review article explains that HLH is a potentially life-threatening condition which can be missed in children and adults; therefore, early diagnosis and effective therapy is required to treat the disease
Stinchcombe et al. [16]	2007	Annual Review of Cell and Developmental Biology	In this article, recent advances on the role of the cytotoxic pathway was discussed which is responsible for lymphocyte hemostasis and immune surveillance	Cytotoxic-mediated activity plays a critical role in the immunological pathway involved in HLH
Popko et al. [17]	2015	Central-European Journal of Immunology	To determine the role of NK cells in the pathogenesis of HLH	There is a role of NK cells in regulation, as well as in cytotoxic abilities in patients with HLH
George et al. [23]	2014	Journal of Blood Medicine	To raise awareness about the understanding of HLH which is responsible for high morbidity and mortality	HLH can be triggered by genetic defects in cytotoxic T cells and NK cells, as well as infections. Treatment including immunosuppression, immune modulation, and chemotherapy plays a pivotal role in disease management
Sen et al. [40]	2016	Indian Journal of Pediatrics	To understand the mechanism of MAS in HLH	This review article explains that MAS is considered a type of secondary HLH. It results from the excessive activation of T-lymphocytes and macrophages leading to this rare syndrome
Verma et al. [44]	2017	Indian Journal of Clinical Biochemistry	To investigate the association between hyperferritinemia and HLH	This case study explains that the measurement of ferritin levels can be used in the diagnosis as well as in disease monitoring of HLH
Cron et al. [45]	2015	Expert Review of Clinical Immunology	To diagnose the clinical features of MAS	This review article explains that the clinical features of MAS include pancytopenia, hyperferritinemia, hepatobiliary dysfunction, and markers of immune activation playing a major role in the pathogenesis of HLH

## Conclusions

HLH is a life-threatening hyperinflammatory condition resulting from the impaired function of CTLs and NK cells, leading to a proliferation of benign hemophagocytic histiocytes. The imbalance between Th1 and Th2 cells is another important mechanism in immune system activation. Because the disease has a high rate of morbidity and mortality, early diagnosis and crucial steps should be taken to improve the overall well-being and survival of HLH patients. In addition, it is one of the most debilitating rare syndromes, which requires comprehensive immunological, clinical, and genetic workups for diagnosis.

HLH poses a diagnostic dilemma for physicians and clinicians because of the nonspecific signs and low incidence. However, the diagnosis can be made by paying imperative attention to the signs and common symptoms of HLH. Still, more research in molecular biology, immunology, and genetics of HLH is needed to explore the effective management and treatment options for all patients who suffer from this rare disorder.
